# Case report: Aortobiiliac bypass with autotransplantation of the horseshoe kidney and renal vascular reconstruction

**DOI:** 10.1016/j.ijscr.2024.110247

**Published:** 2024-09-06

**Authors:** Elena Knochenhauer, Daniel Behrendt, Uwe Zimmermann, Alexandra Busemann, Stephan Kersting

**Affiliations:** a Department of Surgery, Universitätsmedizin Greifswald, Ferdinand-Sauerbruch-Straße, 17475 Greifswald, Germany; b Department of Urology, Universitätsmedizin Greifswald, Ferdinand-Sauerbruch-Straße, 17475 Greifswald, Germany

**Keywords:** Case report, Infrarenal aortic aneurysm, Horseshoe kidney, Renal vascular reconstruction

## Abstract

**Introduction and importance:**

This case report describes the rare encounter of a large infrarenal abdominal aortic aneurysm (AAA) and a horseshoe kidney (HEN), which was managed through comprehensive preoperative planning and effective multidisciplinary collaboration, with the patient being fully informed and consenting to the publication of data and images.

**Case presentation:**

The patient was referred to the Department of Vascular Surgery with increasing abdominal pain. Computed tomography angiography showed that the aneurysm was significantly advanced. For surgical correction of the aortic aneurysm, the horseshoe kidney was autotransplanted intraoperatively to ensure renal vascular reconstruction for further renal perfusion after treatment of the aneurysm.

**Discussion:**

Abdominal aortic aneurysm carries a significant risk of rupture, with more than half of ruptures being fatal before reaching the hospital. When the AAA occurs concomitantly with a HEN, detailed imaging is critical for planning the procedure, as the unusual arterial supply to the HEN complicates the procedure and requires careful strategies to protect renal function while treating the aneurysm.

**Conclusion:**

The treatment of complex cases with severe disease manifestations and difficult anatomy requires careful planning to ensure effective treatment and optimise patient survival and quality of life.

## Highlights

• Coexistence abdominal aortic aneurysm and horseshoe kidney
• Complex renal arterial vascularization
• Autotransplantation of the horseshoe kidney
• Aortobiiliac bypass

## 1 Introduction

With a prevalence of 0.25 %, horseshoe kidney (HEN) is the most common malformation of the genitourinary tract [1,2]. However, coexistence with abdominal aortic aneurysm (AAA) is a rare condition with a prevalence of 0.12–0.15 % [1].

In the present case, we report on the combined presence of a large AAA and a HEN. The treatment in our academic hospital required extensive preoperative planning and good teamwork between radiologists, vascular surgeons, urologists, and anaesthesiologists. The aim was to ensure safe and effective patient care. The work has been reported in line with the SCARE criteria [3].

## 2 Case presentation

A healthy 66-year-old smoker, who had no significant pre-existing conditions on general physical and cardiovascular examination, presented to the vascular surgery consultation in an emergency with acute new onset of increasing abdominal pain associated with a known abdominal aortic aneurysm (AAA). Due to the known AAA, which was found to be progressive in size on sonographic examination last year, the patient is undergoing vascular surgery. In addition, the patient is known to have HEN with complex renal arterial vascularisation, which is why surgical treatment has not been performed to date. Due to the new symptoms, a surgical procedure was now discussed, and an elective operation was scheduled for the near future. Preoperative laboratory tests showed a creatinine level of 84 μmol/l and a GFR of >60 ml/min.

**Fig. 1:**
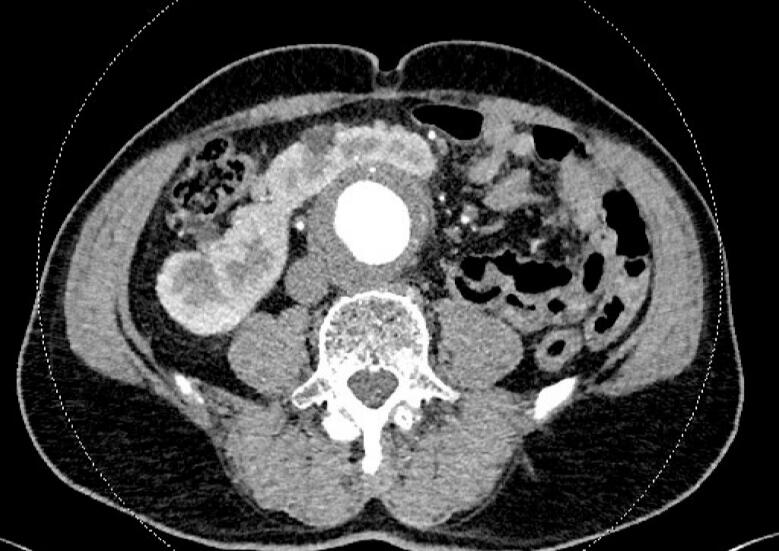
CT morphological position HEN and aorta.

In the computed tomography (CT) angiography performed, the size of the AAA was projected to a maximum diameter of 5.3 × 6.6 cm. The HEN is located loco typico and surrounds the AAA ventrally in a semicircular shape, shown in Fig. 1.

**Fig. 2:**
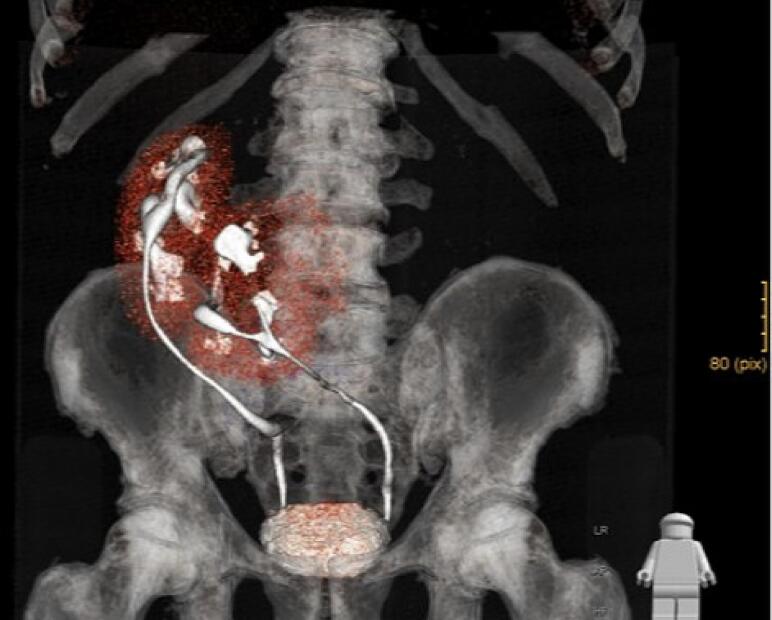
Preoperative CT urogram.

**Fig. 3:**
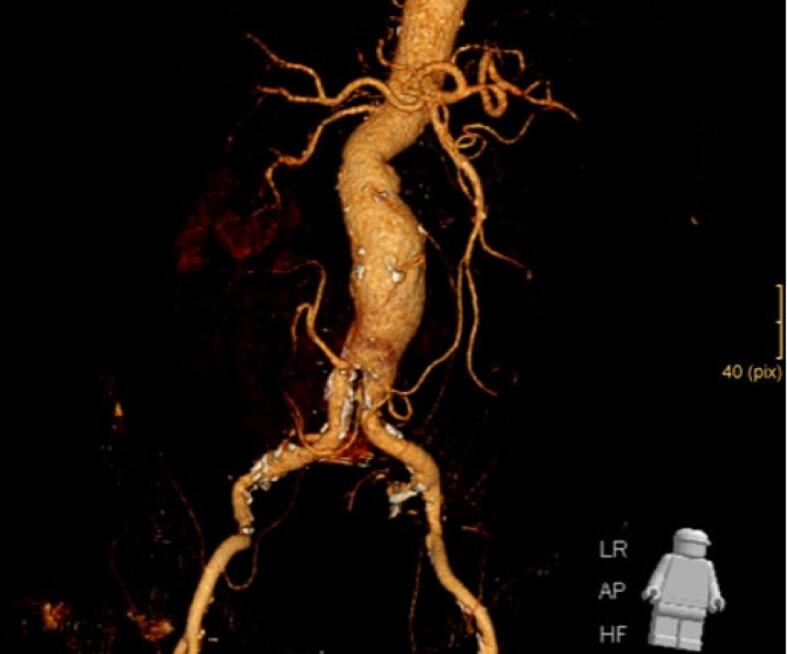
Preoperative CT angiography.

The renal vascular system consisted of a total of six renal arteries, the strongest of which originates directly at the level of the aortic bifurcation. Another one arose directly from the aneurysm at the level of the lumbar vertebral body cover plate four, and two each arose from the right and left common iliac artery. The detailed three-dimensional reconstructions of the CT images of the abdomen and urogenital tract shown in Fig. 2, Fig. 3 enabled intensive preoperative planning. Due to the unique and complex anatomy of the renal vasculature, an open approach was chosen over an endovascular approach.

Intraoperatively, it was difficult to create adequate access to the AAA as it was surrounded ventrally by the HEN in a semicircular shape. The existing renal vasculature and the position of the ureter posed additional challenges for the surgeons during intraoperative mobilization, which is why the decision was made to harvest and auto transplant the HEN. In the further intraoperative course, the right common iliac artery was found to be aneurysmal up to the branch of the external iliac artery. On the left side, the aneurysmosis ended before the iliac bifurcation. Therefore, the decision was made intraoperatively to place an aortobiiliac bypass (Y-prosthesis, Gelsoft 16/8) via end-to-end anastomoses.

**Fig. 4:**
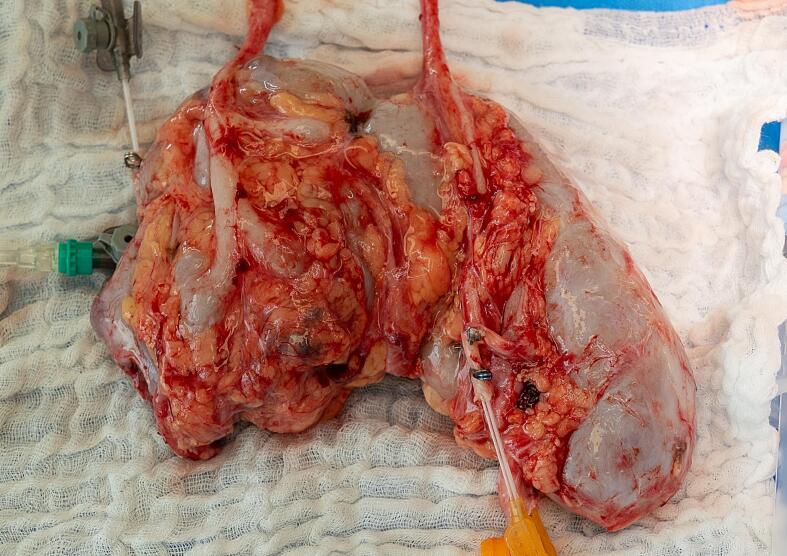
HEN removed intraoperatively.

To ensure rapid revascularisation of the removed kidney, two different surgical teams and surgical sites were used. During this time, the removed kidney was perfused with a Histidine-Tryptophan-Ketoglutarate hypothermia infusion on the urologist's instructions until the blood was completely flushed out. Fig. 4 shows the intraoperatively removed HEN. Subsequently, an arterial reconstruction of the six renal arteries was performed using the removed great saphenous vein (VSM) of the right leg. For this purpose, the two cranial renal arteries were anastomosed end-to-side.

**Fig. 5:**
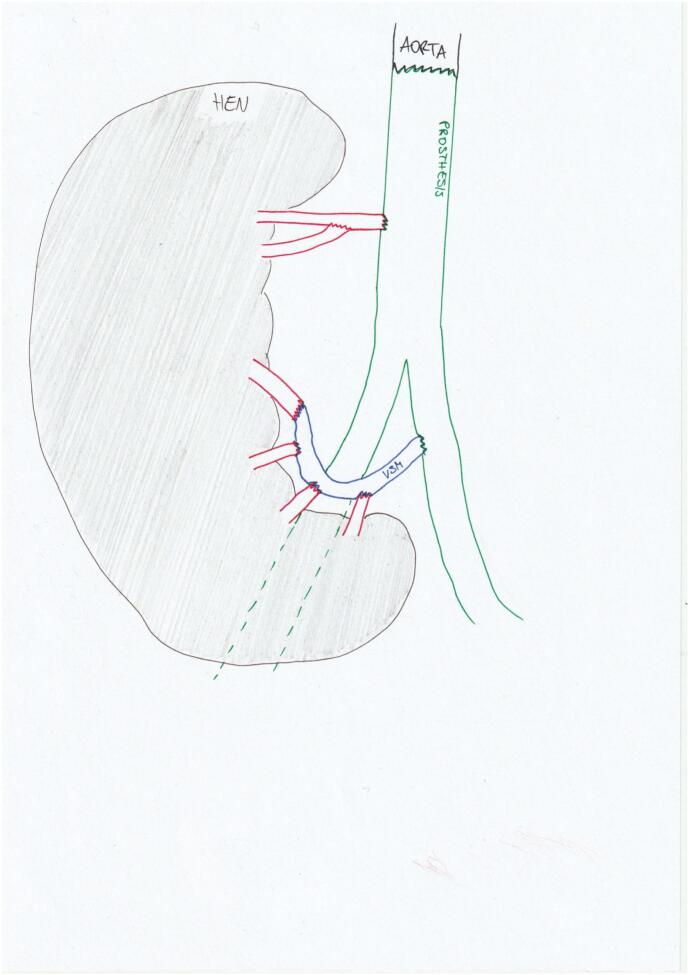
Schematic representation arterial reconstruction.

The remaining 4 renal arteries were anastomosed end-to-end or end-to-side via the great saphenous vein. All anastomoses were sutured with Prolene 5-0 using magnifying glasses. Fig. 5 shows a schematic representation of the arterial vessel reconstruction.

**Fig. 6:**
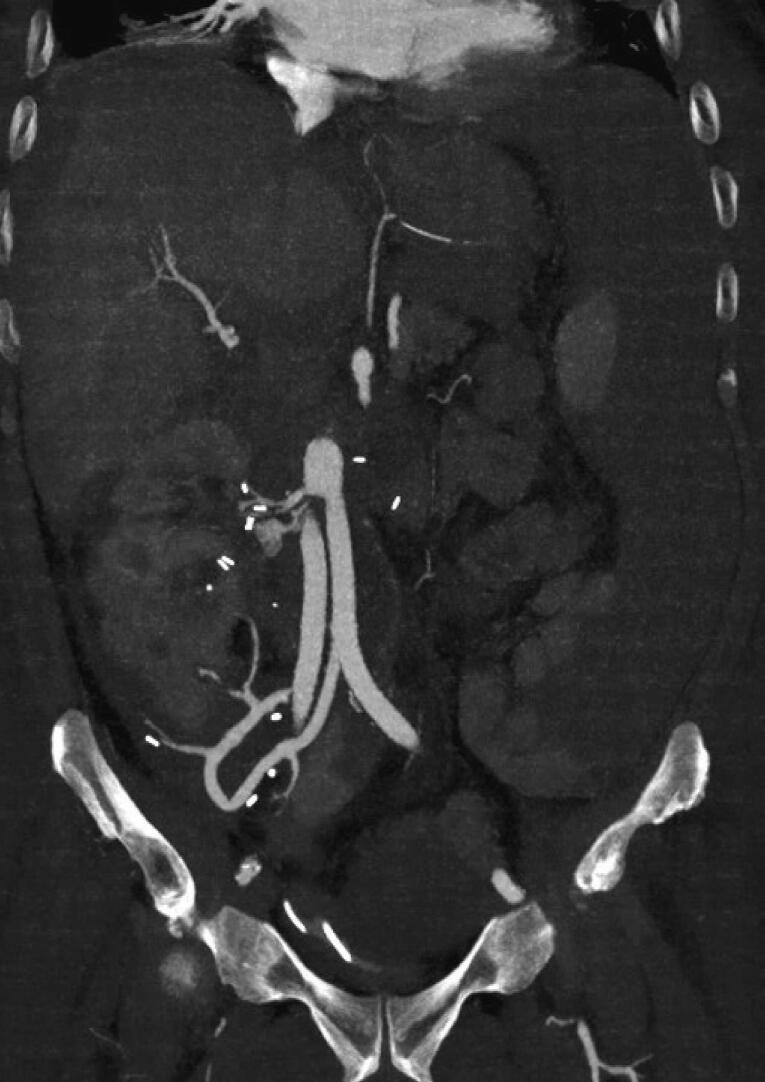
CT morphological arterial reconstruction.

After the orthotopic autoretransplantation, the vascular anastomoses of the renal arteries were performed. Starting with the venous reconstruction, an end-to-side anastomosis was created with the vena cava and the left iliac vein. After clamping the prosthesis, the arterial anastomoses were performed in an end-to-side technique with the main part of the prosthesis and similarly with the left proximal limb of the prosthesis. After the blood flow was released, the renal parenchyma turned pink in colour. The warm ischaemia time was 15 min, the cold ischaemia time after successful Retransplantation was 2 1/2 h. Fig. 6 shows the CT morphological arterial reconstruction after successful Retransplantation.

**Fig. 7:**
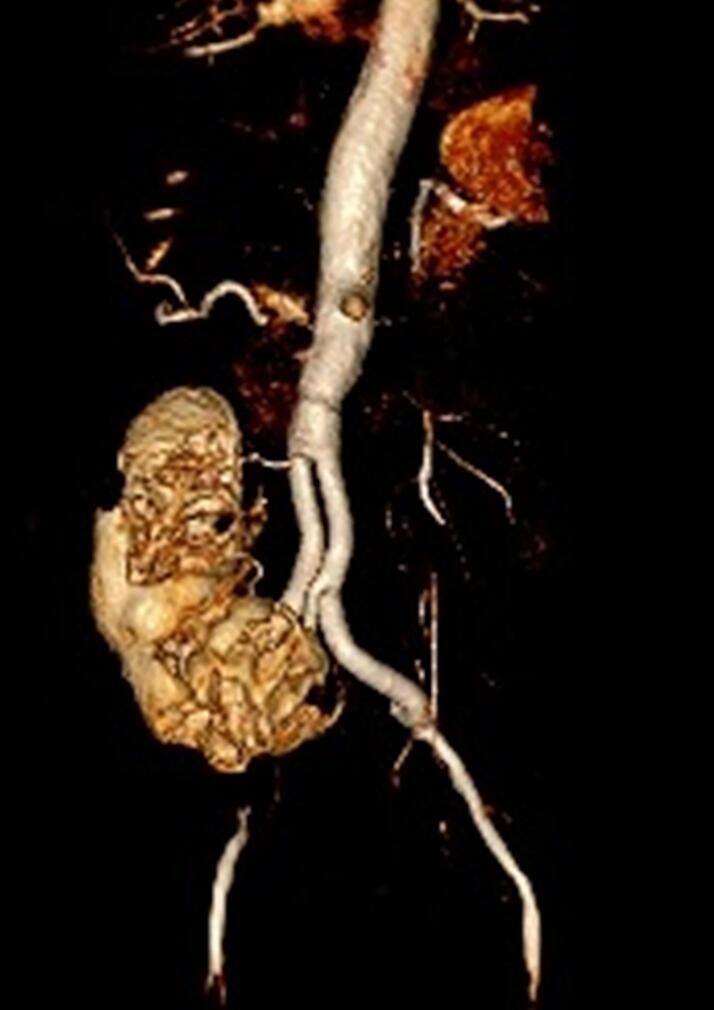
Postoperative magnet resonance angiography (2).

**Fig. 8:**
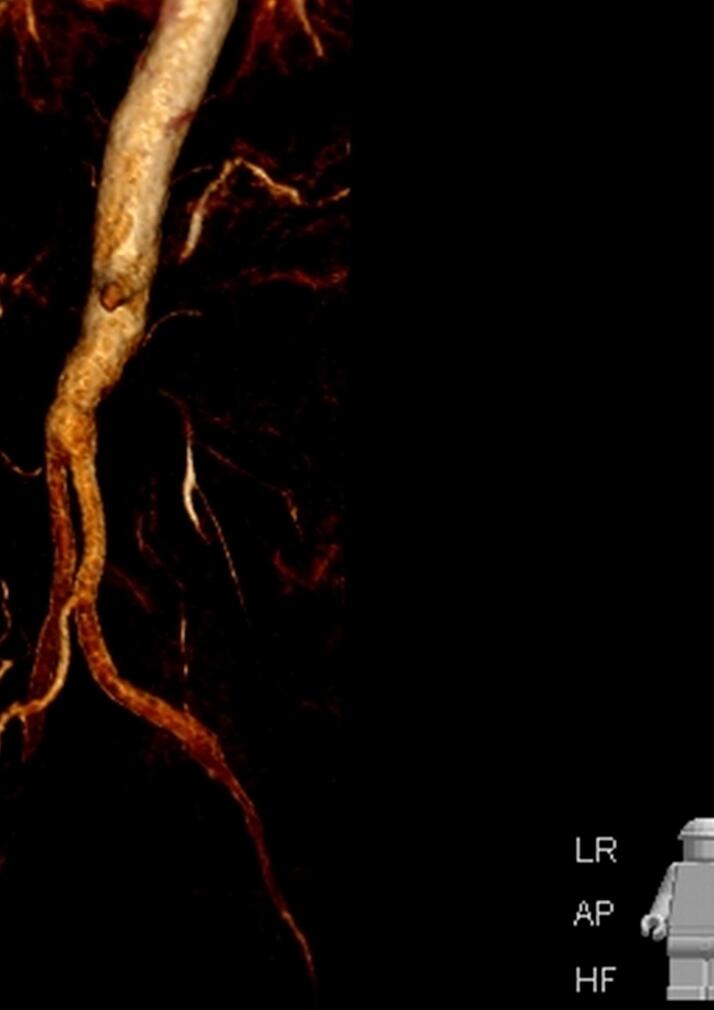
Postoperative magnet resonance angiography.

The Doppler sonographic flow signal of all renal vessels was present after successful revascularization; in addition, both foot pulses were present postoperatively by Doppler sonography. Fig. 7, Fig. 8 show the postoperative vascular reconstruction with the aortic prosthesis in place. In summary, due to the special circumstances, this case represents one of the first described cases in this category.

As planned, the patient was transferred to the intensive care unit after the operation. During this time, renal failure occurred due to the long intraoperative clamping time. Dialysis therapy was therefore initiated postoperatively. With regular excretion during dialysis therapy, the further postoperative course was without complications, so that the patient could be successfully discharged to the rehabilitation clinic.

Here he was again examined for general cardiovascular risk factors and encouraged to maintain an appropriate lifestyle, including abstinence from nicotine.

Sixteen months postoperatively, magnetic resonance imaging showed normal renal perfusion with physiological excretion. The creatinine values are laboratory-chemically restricted, but stable with creatinine values of 240 μmol/l and a GFR of 30 ml/min. The patient has not required dialysis for eight months and enjoys an unrestricted quality of life.

## 3 Discussion

AAA is a life-threatening disease and affects around 3 % of people over the age of 50, with a higher prevalence in men than in women. An important risk factor is nicotine abuse, which not only increases the prevalence but also accelerates the progression of the disease [4].

With a mortality rate of up to 85 %, the most feared complication of AAA is perforation [3]. The mortality of rupture is over 50 %, with most deaths occurring before the patient reaches the hospital. [5] According to the literature, the best diagnostic approach for a symptomatic AAA is either helical CT or conventional CT combined with aortography. This provides the best basis for preoperative planning of the procedure to decide which type of approach and treatment is most appropriate for the patient. [6] In our case, the AAA had a maximum diameter of 6.6 cm in these examinations and had a significantly increased risk of rupture. If the aneurysm had burst, it could have been fatal for the patient.

In the presence of concomitant HEN, further specialized imaging procedures should be performed to obtain precise knowledge of the arterial supply to the kidneys.

The procedure of choice is therefore aortoarteriography [7]. Anomalies of the arterial constitution are the key element of the surgery.

The problem with this anomalous vascular constitution is that the surgeon does not know what function these arteries have. This can even make an aneurysm resection impossible [8]. In contrast, the anomalies of the urinary tract and venous system do not have a significant influence for the surgical procedure.

As renal transplant surgery has shown, these abnormal arterial vascular anomalies are usually end arteries of a renal segment without significant collaterals [9]. Furthermore, horseshoe kidneys are often damaged by stone formation, congestion, or infection. Therefore, resection of the aortic aneurysm should focus on preserving the arterial blood supply to the kidney as much as possible and sparing the functional renal parenchyma as much as possible [8].

In 1984, Crawford et al. determined three relevant basic types of abnormal vascular constitution in HEN, the knowledge of which was decisive for the surgical procedure in >80 % of cases:

Type I: Blood supply is provided by the 2 standard renal arteries above the aneurysm.

Type II: The HEN is supplied by additional arteries from the iliac stroma region below the aneurysm.

Type III: The HEN is mainly or solely supplied by arteries arising from the aneurysm itself [7].

According to the literature, it is usually possible to reconstruct the aorta without affecting any of the type I and II renal arteries [7]. However, type III vascularization is associated with technical difficulties. In our case, it was a type III.

In addition to the location of the vessels, the location of the ureters made it impossible to mobilize the anatomical structures for technical reasons. Intraoperative transection of the isthmus was not performed as it was not possible to clearly separate the renal pelvic caliceal systems in terms of image morphology. Hence the intraoperative decision to perform orthotopic auto transplantation.

Elective extracorporeal renal surgery and renal auto transplantation became popular in the 1970s. It was first performed by Shackman in 1961 in the United Kingdom for renal artery stenosis. Subsequently, in 1962, autotransplantation was performed in the United States for ureteral injuries [10], other applications of this procedure include resection of tumors. Over time, the procedure has also been used to repair complicated renal anomalies, as conventional techniques have not always been able to address the wide range of problems [11].

In recent studies, the success rate of transplants with at least 20 autotransplants is between 72 % and 92 % for malignancy. The symptom success rate, which is defined by the cure or improvement of hypertension, improvement of flank pain or reduction of urinary tract infections, ranges from 59 % to 94 % for renovascular vascular disease [10].

In their 2000 publication, Stroosma and colleagues describe 31 case reports of horseshoe kidney transplantation between 1975 and 1998, but 48 recipients were involved. Of these, 10 HEN were transplanted en bloc and 21 HEN were divided and transplanted into 38 recipients. With an average follow-up time of 22 months, the overall success rate for HEN transplantation was 87 %. As explicit autotransplantation of HEN is rare, there are very few documented cases in the literature. Due to the fact that this procedure is usually performed in special clinical situations, it is difficult to make an exact statement about the frequency [12].

Better visualization of intraparenchymal pathology, a bloodless working field and a significantly shortened warm ischemia time are the potential technical advantages offered of this approach [13]. Results of autografting show a graft loss rate of 4 %, most often caused by vascular thrombosis or due to primary nonfunction despite sufficient perfusion [14].

The additional intraoperative problem in the presence of HEN is the correlative position of the isthmus with the position of the infrarenal AAA as well as the position of the renal arteries [15]. Embryologically, abnormal fusion of the ureteral buds occurs during in the fourth to sixth week of gestation due to fusion of the inferior poles of the metanephric masses. This impedes the rotation and ascent of the renal masses into the lateral renal pits, creating a horseshoe around the inferior mesenteric artery [16]. This spatial overlap makes surgical access and the resection itself considerably more difficult.

Finally, a few limitations of this case report should be mentioned. Due to the rarity of this combination of diseases, it is difficult to transfer this case to the wider patient population. The informative value for general clinical practice is limited. In addition, there is no comparison with other treatment approaches or similar cases without HEN autotransplantation. Furthermore, there is very little literature on the long-term results after HEN autotransplantation. Inter-individual differences between patients should also be noted. Each patient has anatomical, physiological and genetic characteristics that always influence the outcome.

## 4 Conclusion

Complex cases like this, with two extreme forms of disease manifestation and a difficult anatomy, require good and close cooperation in a multidisciplinary team. Very good communication and cooperation between the specialties is required to ensure appropriate treatment. Survival and an adequate quality of life for the patient are the definitive treatment goals. Through a precise prioritization of the therapeutic approach as well as careful planning and structured work, these goals can be emphatically achieved.

## Ethical approval

No ethics approval was required for this case report from our ethics committee, the ethics committee at Greifswald University Medical Centre. Case reports are not regarded as research at our university medical centre. The patient was informed in detail and the procedure was discussed in detail. The patient gave his full consent to the procedure. This is available in written form.

## Sources of funding

N/A.

## Author contribution

Operation procedure DB, UZ, AB, EK; original draft writing EK, DB; review and editing UZ, AB, SK; supervision SK.

## Guarantor

Elena Knochenhauer, Ferdinand-Sauerbruch-Straße, 17475 Greifswald, Germany. elena.knochenhauer@uni-greifswald.de.

## Registration of research studies

N/A.

## Consent

Written informed consent was obtained from the patient for publication of this case report and accompanying images. A copy of the written consent is available for review by the Editor-in-Chief of this journal on request.

## Declaration of competing interest

The authors have no competing interests.
